# Antimicrobial and antioxidant potential of different solvent extracts of the medicinal plant *Geum urbanum* L.

**DOI:** 10.1186/s13065-017-0343-8

**Published:** 2017-11-07

**Authors:** Lyudmila Dimitrova, Maya M. Zaharieva, Milena Popova, Nedelina Kostadinova, Iva Tsvetkova, Vassya Bankova, Hristo Najdenski

**Affiliations:** 1 Department of Infectious Microbiology, The Stephan Angeloff Institute of Microbiology, Bulgarian Academy of Sciences, Acad. G. Bonchev Str. Bl. 26, 1113 Sofia, Bulgaria; 2 Institute of Organic Chemistry with Centre of Phytochemistry, Bulgarian Academy of Sciences, Acad. G. Bonchev Str. Bl.9, 1113 Sofia, Bulgaria

**Keywords:** *Geum urbanum* L., Plant extracts, Antibacterial activity, Minimal inhibitory concentration, Radical scavenging activity, Phenolics

## Abstract

Many *Geum* species are known to be rich in biologically active compounds and therefore could be a source of new natural products with pharmacological potential. The medicinal plant *Geum urbanum* L. is widespread in Bulgaria and has been used in folk medicine. In the present study, the methanol extracts of the roots and aerial parts of *G. urbanum* and their fractions (petroleum ether, ethyl acetate and *n*-butanol) were investigated for antibacterial and radical scavenging activity. The ethyl acetate and *n*-butanol fractions inhibited the growth of Gram-positive pathogenic and opportunistic bacteria from the genus *Staphylococcus* (MIC EtOAc: 0.078 mg/ml aerial and 0.156 mg/ml roots; MIC *n*-BuOH: 0.156 mg/ml aerial and 1.25 mg/ml roots) and the species *Bacillus cereus* stronger than the other extracts and fractions tested (MIC EtOAc: 0.078 mg/ml aerial and 0.156 mg/ml roots; MIC *n*-BuOH: 0.156 mg/ml aerial and 0.078 mg/ml roots), and showed corresponding radical scavenging activity (EtOAc: EC_50_ 1.5 µg/ml aerial, 0.8 µg/ml roots; *n*-BuOH: 4.5 µg/ml aerial; 3.7 µg/ml roots). Additionally, their total phenolic content was quantified (% of dry EtOAc fractions of roots 61%, of arial parts 32%; of dry *n*-BuOH fractions of roots 16%, of arial parts 13%). Seven compounds were isolated and identified spectroscopically from the ethyl acetate extract. Two acetylated ellagic acid rhamnosides were found for the first time in the genus *Geum* and three others, tormentic acid, niga-ichigoside F1, and 3,3′-di-*O*-methylellagic acid-4-*O*-*β*-d-glucopyranoside, were newly detected for the species *G. urbanum.* Our results reveal that *G. urbanum* L. is a perspective medicinal plant and deserves further, more detailed studies.

**Electronic supplementary material:**

The online version of this article (10.1186/s13065-017-0343-8) contains supplementary material, which is available to authorized users.

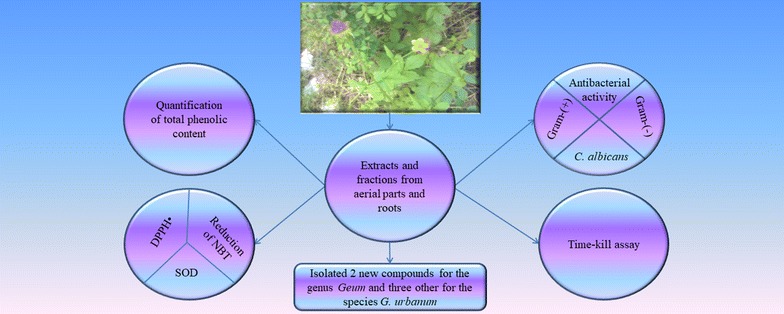

## Introduction

The genus *Geum* (Rosaceae) consists of about 70 plant species distributed in temperate regions [1]. Many *Geum* species are rich in biologically active compounds and therefore could be a source of new plant products with pharmacological potential. Eight of them are part of the Bulgarian flora [2], among which the medicinal plant *Geum urbanum* L. is widespread over the territory of the country. This herbaceous perennial plant species commonly known as wood avens or St. Benedict’s herb [3] has been recommended since ancient times in the folk medicine for treating of gastro-intestinal diseases, disorders of the liver, biliary tract and uterus, as well as against hemorrhoids [4, 5]. The roots and rhizomes decoction has been applied for the treatment of diarrhoea, dysentery, dyspepsia, gastroenteritis, whereas the aerial parts infusion was used in cases of leucorrhoea, haemorrhages and fever [4]. The infusion is ingested against rheumatism, gout, infections and fever [6]. The ethnopharmacological data suggest antimicrobial and radical scavenging properties of the extracts. It is well established that infectious diseases can provoke oxidative stress events in the human body, because reactive oxygen and nitrogen radicals secreted by bacterial pathogens accumulate in the microenvironment of affected tissues. In this case, the antioxidant system of the human body could not act adequately to prevent various oxidative damages. Some studies have even suggested that bactericidal antibiotics may increase the oxidative stress via the Fenton reaction, though this finding remains controversial [7]. Therefore, one of the important points today is to focus on investigation of plants that not only possess strong antioxidant properties but also exhibit antimicrobial activity [8, 9].

More than 200 compounds (monoterpenoids, sesquiterpens, triterpenoids, flavonoids, hydrolysable tannins, phenylpropanoids and others) have been isolated from the genus *Geum* after 1920 [1], but the studies in chemical composition of *G. urbanum* are limited: a few articles have been published on the isolation of chemical constituents of *G. urbanum*. From the roots, the rare disaccharide vicianose [10], the phenylpropanoid gein [11], catechin, gallic acid, galloylglucose, caffeic acid, chlorogenic acid and ellagic acid [12] were identified. More recently, ellagitannins and procyanidins were isolated from roots of *G. urbanum* [3, 13]. Essential oils from aerial and underground parts of the plant have also been studied [14].

To our knowledge, data concerning the antimicrobial and radical scavenging potential of *G. urbanum* in relation to its chemical composition have not been reported. Thus, in the present study we aimed to investigate the antibacterial and radical scavenging activity of extracts and fractions obtained from aerial and underground parts of *G. urbanum*, to determine the total phenolic content and to isolate some individual chemical compounds.

## Materials and methods

### Plant material

Dry roots and aerial parts from *Geum urbanum* were commercial products, produced by Sunny-Yambol, Ltd^®^, according to the lable it was collected in April 2014 from district of Stara Zagora, Bulgaria.

### Extraction and solvent fractionation

Five hundred g roots and 500 g aerial parts of *G. urbanum* were extracted by maceration each in 3 l methanol for 2 days at room temperature, the extracts were filtered and the extraction was repeated. The total MeOH extracts were concentrated in vacuo, and extracted successively with petroleum ether, ethyl acetate (EtOAc) and *n*-butanol (*n*-BuOH). The fractions obtained from aerial parts were evaporated to give 7.9 g petroleum ether; 10.4 g EtOAc and 17.82 g *n*-BuOH dry residue, and from roots, 1.9 g petroleum ether; 14.1 g EtOAc and 14.8 g *n*-BuOH dry residue. Parts of the total MeOH extracts was evaporated to dryness and used in other experiments.

### Antibacterial activity

#### Test microorganisms

The test bacteria used for antimicrobial susceptibility testing were: *Staphylococcus aureus* NBIMCC 3359 (National Bank for Industrial Microorganisms and Cell Cultures, Bulgaria), *Staphylococcus aureus* ATCC 3865 (American Type Cell Culture Collection, USA), methicillin-resistant *Staphylococcus aureus* (MRSA) NBIMCC 8327*, Staphylococcus epidermidis* NBIMCC 1093, *Streptococcus pyogenes* SAIM 10535 (Collection of the Stephan Angeloff Institute of Microbiology, Bulgaria), *Bacillus cereus* ATCC 9634, *Bacillus subtilis* SAIM 1A95, *Listeria monocytogenes* SAIM C12, *Escherichia coli* SAIM WF+, *Pseudomonas aeruginosa* NCTC 6749 (National Collection of Type Cultures, England), *Salmonella typhimurium* SAIM 123 and *Candida albicans* SAIM 562.

#### Culture medium and growth conditions

For each bacterium used in this study Muller Hinton agar (MHA) and broth (MHB) (CM0337B, resp. CM0405B, Thermo Scientific-Oxoid, UK) were applied. Sabouraud-Glucose agar supplemented with gentamicin (40 μg/ml) (CM0041, Oxoid, Basingstoke, UK) was used as culture medium for *C. albicans.* All microorganisms were grown at 37 °C overnight except *B. cereus* ATCC 9634, which was grown at 30 °C.

#### Minimal inhibitory (MIC) and bactericidal (MBC) concentrations

The antimicrobial activity was estimated by the broth microdilution method according to CLSI procedures [15] as published before [16]. Briefly, bacterial inoculums with concentration 10^5^ CFU/ml were added to microtitre trays containing MHB loaded with *G. urbanum* MeOH extracts and fractions or single compounds in concentrations varying from 0.039 to 2.5 mg/ml. Plates were incubated at 37 °C for 18 h. The negative control was prepared by spreading 10 μl of the inoculation-suspension on a nutrient agar plate and incubated at 37 °C overnight. Gentamicin was used as reference antibiotic according to the requirements of EUCAST. Experiments were performed in triplicate. MIC were determined visually as the lowest concentration without visible growth [17]. MBC were determined by overnight incubation on MHA of 100 µl from the untreated control and samples treated with ½ × MIC, MIC and 2 × MIC for further 18 h at 37 °C. MBC were read as concentrations where no bacterial growth occurred on the agar plates [17].

#### Dehydrogenase (DEHA) activity

The DEHA activity of the test microorganisms was assessed by spectrophotometric analysis [18]. For the latter treated and untreated bacterial cells were incubated for 60 min at 37 °C with MTT dye (3-(4,5-dimethylthiazolyl-2)-2,5-diphenyltetrazolium bromide, M2128-1G, Sigma-Aldrich) in final concentration 0.05 mg/ml. Formazan crystals were dissolved by an equivalent volume of 5% HCOOH in isopropanol. Absorption was measured with an ELISA reader (BioTek Elx800, USA) at 550 nm (reference 690 nm) against a blank solution. As far as some of the tested extracts, fractions and compounds possess polyphenolic hydroxyl groups reducing the MTT dye [19–21], their own absorbance was measured in parallel, in the absence of bacterial inoculum.

#### Time-kill effect

Assays for the rate of killing effect was carried out for EtOAc fractions from roots and aerial parts against *B. cereus* ATCC 9634 by using a protocol of Olajuyigbe and Afolayan [22]. overnight culture (10 ml MHB) was spectrophotometric measurement at 600 nm and diluted to 10^5^ CFU/ml bacteria concentration. The experiment was performed into 96-well microplate. The bacterial inoculums were added to each well containing MHB loaded with *G. urbanum* EtOAc fractions in concentrations 2.5, 1.25 and 0.625 mg/ml. The final volume of each sample was 100 µl/well. The plate was incubated at 30 °C. 100 µl aliquot was transferred from each well onto petri dishes with 20 ml MHA at 0, 4, 12 and 24 h and incubated at 30 °C overnight.

### Radical scavenging activity

#### DPPH• assay

Each extract and fraction was evaluated for the radical scavenging ability for using of the bleaching level of purple colored solution of 1,1-diphenyl-2-picrylhydrazyl (DPPH•)-stable radical used as a reagent, according to the method of Murthy et al. [23] with small modifications. Various concentrations of the plant extracts and fractions were added to 1 ml of DPPH• (100 µM) solution in ethanol. The absorbance was read against a blank at 517 nm, after 30 min incubation period at 37 °C. Inhibition of free radical DPPH• in percent was calculated according to the formula:

$${\text{Inhibition}} \% \, = \, \left( {{\text{A}}_{\text{blank}} \,{-}\,{\text{A}}_{\text{sample}} /{\text{A}}_{\text{blank}} } \right)\, \times \, 100,$$

where A_blank_ is the absorbance of the control reaction (containing all the reagents except the test compound) and A_sample_ is the absorbance of the test compound. The concentration of the MeOH extracts and fractions providing 50% inhibition (IC_50_) was calculated on the basis of graph plot-inhibition percentage against extract or fraction concentration (0.5, 1.0, 2.5, 5.0, 10.0, 25.0, 50.0 µg/ml). Caffeic acid was used as a positive control.

#### Superoxide anion scavenging activity

Determination of superoxide anion scavenging activity was done by inhibition of nitro blue tetrazolium (NBT) reduction by photochemically generated O_2_^−^ [24]. Samples were prepared to contain 5 µg/ml of the corresponding preparations (Fig. 3). The reaction mixture contained 56 μM NBT, 0.01 M methionine, 1.17 μM riboflavin, 20 μM NaCN and 0.05 M phosphate buffer with a pH of 7.8. Superoxide presence was evaluated by the increase in absorbance at 560 nm at 30 °C after 6 min of incubation from the beginning of the illumination. The dose-dependence of the superoxide anion scavenging effect of extracts, fractions and caffeic acid (reference substance) was calculated against different concentrations (1, 2, 3, 4, 5, 10, 25, 50 µg/ml). All values were the mean of three measurements and expressed as mean ± SD.

### Quantification of total phenolics

To determine the amount of polyphenol compounds in MeOH extracts 1 g dry mass was extracted twice with MeOH, 1:10 (w/v) under ultrasound conditions at 50 °C for 1 h. The extracts obtained after filtration were combined in a 25 ml volumetric flask and diluted with MeOH. Dry extracts of the petroleum ether (50 mg), EtOAc (50 mg) and *n*-BuOH (200 mg) fractions were dissolved in MeOH in 25 ml volumetric flasks. For every extract and fraction, the procedures were performed in triplicates.

An aliquot (3 ml), were transferred in a 25 ml volumetric flask and diluted with MeOH except of petroleum ether fraction which were diluted to 5 ml. All final solutions were subjected to spectrophotometric analysis.

Methanolic solutions of gallic acid (from 0.2 to 0.0125 mg/ml) were used to generate the standard curve. To 5 ml distilled water, 0.5 ml of the standard solution were added, after that 2 ml of Folin–Ciocalteu reagent and 3 ml of 20% Na_2_CO_3_ were added and the volume made up to 25 ml (volumetric flask). After 2 h the absorbance was measured at 760 nm (blank prepared in the same way, 0.5 ml of MeOH instead of standard solution). For the analysis of the plant extracts and their fractions, 0.5 ml of the corresponding solution was applied in the same procedure. Every analysis was performed in triplicate.

### Isolation of chemical constituents of EtOAc fraction from roots

Fourteen grams of EtOAc fraction from the roots were subjected to vacuum liquid chromatography on silica gel using a CHCl_3_–MeOH gradient system to give 12 subfractions (A–L). Subfractions from B to E were combined in BE (1.35 g) and subjected to column chromatography (CC) on silica gel using a CHCl_3_–MeOH gradient system to give 15 subfractions (BE1–BE15). Subfraction BE5 was subjected to CC on Sephadex LH-20 using a CHCl_3_–MeOH gradient system to obtain tormentic acid **1** (8.6 mg) [25]. Subfractions BE8–BE10 were combined (70 mg) and subjected to preparative thin-layer chromatography (TLC) with EtOAc–MeOH (25:1) as a mobile phase to obtain two compounds: 3-*O*-methylellagic acid-3′-*O*-*α*-3″-*O*-acetylrhamnopyranoside **2** (14 mg) and 3-*O*-methylellagic acid-3′-*O*-*α*-2″-*O*-acetylrhamnopyranoside **3** (15 mg) [26].

Subfractions F and G were combined in FG (4.39 g) to give 26 subfractions. Subfraction FG9 was subjected to CC on silica gel using a CHCl_3_–MeOH–H_2_O gradient system to give 13 subfractions. Subfraction FG9-3 was subjected to preparative TLC with EtOAc–MeOH (15:1) as a mobile phase to obtain cathechin **4** (6.4 mg) [27]. Subfractions from FG9-7 to FG9-12 were combined (20 mg) and subjected to CC on silica gel using a CHCl_3_–MeOH gradient system to obtain 3,3′-di-*O*-methylellagic acid-4-*O*-*β*-d-glucopyranoside **5** (3.8 mg) [28]. Subfraction FG11 was subjected to CC on Sephadex LH-20 using MeOH as a mobile phase to give 9 subfractions. Subfraction FG11-1 was purified by CC on Sephadex LH-20 to obtain niga-ichigoside F1 **6** (31 mg) [27]. Subfraction FG12 was subjected to CC on Sephadex LH-20 using MeOH as a mobile phase to give 10 subfractions. Subfraction FG12-2 was gein **7** (31.4 mg) [27].

**Fig. 1:**
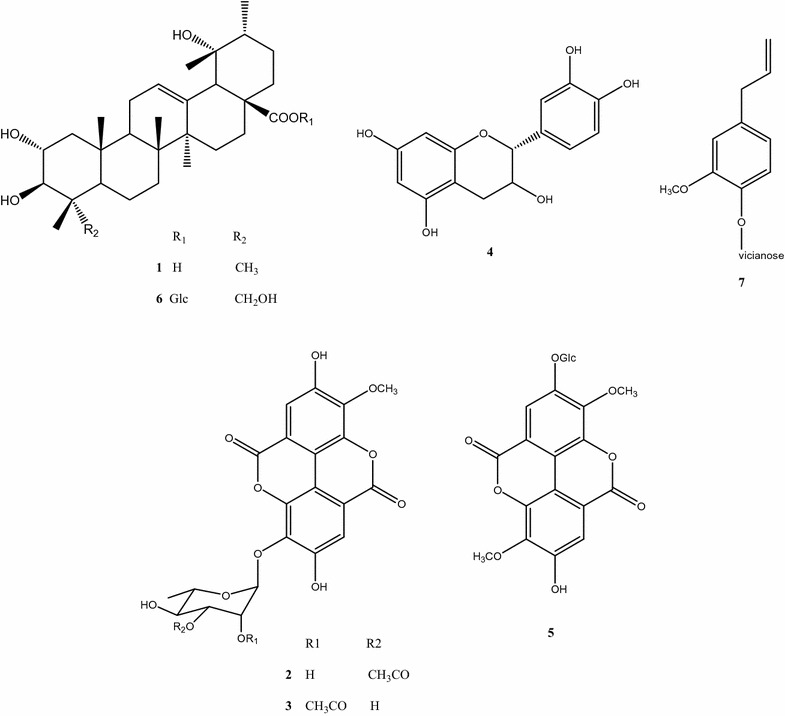
Structures of the compounds isolated from *G. urbanum*

The structures of the isolated compounds, tormentic acid **1**, 3-*O*-methylellagic acid-3′-*O*-*α*-3″-*O*-acetylrhamnopyranoside **2**, 3-*O*-methylellagic acid-3′-*O*-*α*-2″-*O*-acetylrhamnopyranoside **3**, catechin **4**, 3,3′-di-*O*-methylellagic acid-4-*O*-*β*-d-glucopyranoside **5**, niga-ichigoside F1 **6**, and gein **7**, (Fig. 1) were identified by means of NMR spectral data (1D and 2D) and comparison with literature data. All chemicals and solvents were of analytical grade. NMR spectra were recorded on Bruker AV 600 spectrometer (600 MHz for ^1^H and 150 MHz for ^13^C).

## Results

### Antibacterial activity

#### MIC, MBC and DEHA activity

MeOH extracts and petroleum ether, EtOAc and *n*-BuOH fractions of the MeOH extracts from underground and aerial parts of *G. urbanum* were investigated. All extracts and fractions displayed varying antibacterial activity against *S. aureus* NBIMCC 3359*, S. aureus* ATCC 6538 P, MRSA NBIMCC 8327, *S. epidermidis* NBIMCC 1093 and *B. cereus* ATCC 9634 in concentration range 0.039–2.5 mg/ml. No activity was found against *L. monocytogenes* SAIM C12, *S. pyogenes* SAIM 10535, *B. subtilis* SAIM 1A95, *E. coli* SAIM WF+, *P. aeruginosa* NCTC 6749, *S. typhimurium* SAIM 123 and *C. albicans* SAIM 562. The results are listed in Table 1.

**Table 1:** Minimal inhibitory and bactericidal concentration and dehydrogenase activity of MeOH extract and its fractions from *G. urbanum*

Test-bacteria	Parameters	Extracts/fractions								Control
		MeOH		Petroleum ether		EtOAc		*n*-BuOH		
		Aerial parts	Roots	Aerial parts	Roots	Aerial parts	Roots	Aerial parts	Roots	
*S. aureus* NBIMCC 3359	MIC (mg/ml)	0.625	2.5	2.5**	2.5	0.078	0.156	0.156	1.25	Gentamicin (mg/L) 0.0312
	DEHA^a^ activity (%) ± STD	8.8 ± 2.2	20 ± 7.6	15 ± 1.0	15.8 ± 1.1	20.8 ± 2.2	47.5 ± 2.4	58.6 ± 3.4	47.9 ± 3.2	
	MBC (mg/ml)	> 2.5	> 2.5	> 2.5	> 2.5	> 2.5	> 2.5	> 2.5	> 2.5	
*S. aureus* ATCC 6538 P	MIC (mg/ml)	0.625	2.5	1.25	1.25	0.313	1.25	0.313	0.625	Gentamicin (mg/L) 0.125
	DEHA activity (%) ± STD	2.6 ± 0.6	54.1 ± 2	14 ± 1.0	15 ± 1.0	21.5 ± 2.6	34.9 ± 2.7	41 ± 0.09	42.2 ± 5.7	
	MBC (mg/ml)	> 2.5	> 2.5	> 2.5	> 2.5	> 2.5	> 2.5	> 2.5	> 2.5	
Methicillin resistant *S. aureus* NBIMCC 8327	MIC (mg/ml)	2.5	> 2.5	> 2.5	0.313	0.039	0.313	0.156	0.156	Gentamicin (mg/lL) 0.125
	DEHA activity (%) ± STD	28.1 ± 0.1	–	–	4.2 ± 0.3	16.6 ± 1.1	6.9 ± 0.4	40.6 ± 2.0	37 ± 0.4	
	MBC (mg/ml)	> 2.5	> 2.5	> 2.5	> 2.5	> 2.5	> 2.5	> 2.5	> 2.5	
*S. epidermidis* NBIMCC 1093	MIC (mg/ml)	2.5	> 2.5	> 2.5	1.25	0.039	1.25	0.078	0.156	Gentamicin (mg/L) 0.0312
	DEHA activity (%) ± STD	25.4 ± 1.9	–	–	15.23 ± 1.9	14.05 ± 2.7	47 ± 1.4	40.9 ± 7.1	38 ± 5.4	
	MBC (mg/ml)	> 2.5	> 2.5	> 2.5	> 2.5	> 2.5	> 2.5	> 2.5	> 2.5	
*B. cereus* ATCC 9634	MIC (mg/ml)	2.5	2.5	> 2.5	2.5	0.078	0.156	0.078	0.078	Gentamicin (mg/L) 0.125
	DEHA activity (%) ± STD	31 ± 0.5	39.9 ± 1.8	–	30.3 ± 1.3	28.9 ± 0.05	26.2 ± 1.4	45.7 ± 2.8	51.4 ± 2.4	
	MBC (mg/ml)	> 2.5	> 2.5	> 2.5	> 2.5	0.625	0.625	> 2.5	> 2.5	
	DEHA activity (%) ± STD	–	–	–	–	0	1.9 ± 0.5	–	–	

** 2.5 mg/ml is the higher test-concentration

^a^DEHA (dehydrogenase) activity test refers to the enzyme NADH

The lowest MIC values were demonstrated by the EtOAc fractions from aerial and parts and roots of the plant (0.039–1.25 mg/ml) against five strains Gram-positive test bacteria. Petroleum ether fractions had the lowest activities against test bacteria (1.25–2.5 mg/ml and more). Additionally, the values of respiratory activity were determined for all MIC and MBC. Aiming to detect the metabolic activity of bacteria treated with different fractions, a MTT assay was performed. The DEHA activity test is based on the principle that DEHA enzymes are produced by all living cells and this assay can be related to the number of live cells present [29] (results in Table 1). The EtOAc fractions showed lowest value of respiratory activity against *B. cereus* ATCC 9634 (0–1.9%) in concentrations 0.625 mg/ml. In cases where no inhibitory or bactericidal effect was established for the respective extract or fraction, the results of respiratory activity were equivalent to untreated control and were therefore not shown in Table 1.

#### Time-kill effect

In order to determine the microbicidal effect of the most active EtOAc fractions, a time-kill assay was performed in vitro. The results are presented in Table 2.

**Table 2:** In vitro time-kill assay of EtOAc fractions from aerial parts and roots of *G. urbanum* against *B. cereus* ATCC 9634

Intervals (h)	EtOAc fractions					
	Roots (log_10_)			Aerial parts (log_10_)		
	MBC	2 × MBC	4 × MBC	MBC	2 × MBC	4 × MBC
0	3.975	3.997	4.003	4.073	4.015	4.019
4	3.934	3.657	3.155	3.986	3.659	3.365
12	3.459	3.167	1.845	3.719	3.288	2.954
24	2.550	1.398	–	3.681	3.097	1.602

Data are presented in terms of the log_10_ CFU/ml change and are based on the conventional bactericidal activity standard, that is a 3 log_10_ CFU/ml or greater reduction in the viable colony count [30]. Average log reduction in viable cell count in time-kill assay ranged between 4.019 log_10_ to 1.6 log_10_ CFU/ml after 24 h of treatment with 4 × MBC with EtOAc fraction from aerial parts and 4.003 log_10_ to zero after 24 h of treatment with 4 × MBC with EtOAc fraction from roots. Growth inhibition and efficacy of the EtOAc fractions were observed to be dose dependent and time dependent, producing distinct time-kill profile for *B. cereus* ATCC 9634.

### Radical scavenging activity

#### DPPH• radical scavenging activity

**Fig. 2:**
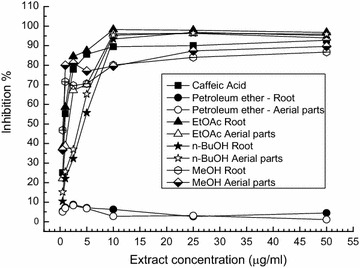
Free radical scavenging effect of different MeOH extracts and fractions from *G. urbanum* (DPPH• assay)

The radical scavenging potential of *G. urbanum* was evaluated by two complementary methods. The free radical scavenging activity, determined by the DPPH• assay, is visualized in Fig. 2. Among all tested extracts, the best scavenging activity was demonstrated for the roots and the aerial parts EtOAc fractions: the values were close to that of the antioxidant agent caffeic acid used as a positive control. Their EC_50_ values were, respectively, 0.8 and 1.5 µg/ml. The inhibitory potency of *n*-BuOH fractions was next in line (with EC_50_ of 4.5 and 3.7 µg/ml for the aerial parts and roots, respectively). The potency of total MeOH extracts followed. The inhibitory effect of the petroleum ether fractions increased only up to 2.5 µg/ml of concentration.

#### Superoxide anion radical scavenging activity

**Fig. 3:**
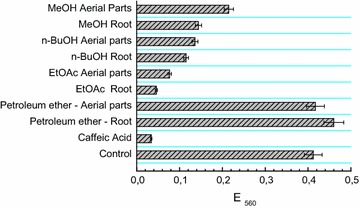
Inhibitory effect of the plant extracts and fractions from *Geum urbanum* on the reduction of NBT by photochemically generated superoxide anion radicals

Additional investigation of the antiradical activity of the MeOH extracts from *G. urbanum* and the fractions thereof was performed in a non-enzymatic system: NBT, methionine and riboflavin. Under these conditions, superoxide anion radicals were generated photochemically. Most of the studied extracts inhibited the development of the color, produced during the reaction between O_2_^−^ and NBT. The highest scavenging activity was demonstrated by EtOAc fractions from roots and aerial parts, followed by *n*-BuOH fractions and then by total MeOH extracts. The results are visualized in Fig. 3.

**Fig. 4:**
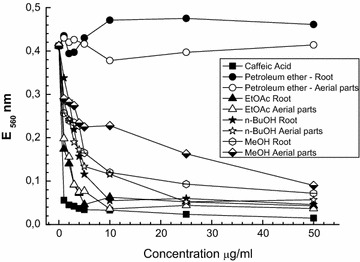
Dose-dependence of the superoxide anion scavenging effect of the MeOH extracts and fractions from *Geum urbanum*

Moreover, the studied extracts and fractions suppressed the release of the superoxide anion radical in a dose-dependent manner (Fig. 4). The 50% O_2_^−^ scavenging concentrations (IC_50_) of EtOAc fractions from roots and aerial parts were found to be 0.9 µg/ml, and of the positive control caffeic acid 0.7 µg/ml. On the other hand, both petroleum ether fractions showed no significant changes in O_2_^−^ scavenging activity when applied in the incubation mixture.

### Quantification of total phenolics

The concentration of the total polyphenolic compounds was determined in MeOH extracts, petroleum ether, EtOAc and *n*-BuOH fractions by the Folin–Ciocalteu method.

The highest content of polyphenol compounds was found in the EtOAc fractions from roots and aerial parts, followed by *n*-BuOH and petroleum ether fractions (Table 3). The two petroleum ether fractions contained very low amounts of polyphenolic compounds—from 1 to 2.8%. It is obvious that EtOAc is the best solvent to extract polyphenols from the total MeOH extracts.

**Table 3:** Total phenolic content (% of dry extract) in different extracts and fractions of *G. urbanum* L.

Extracts/fractions	Total phenolics	
	Roots	Aerial parts
MeOH	19 ± 4	11.3 ± 0.6
Petroleum ether	2.8 ± 0.3	1.0 ± 0.3
EtOAc	61 ± 9	32 ± 3
*n*-BuOH	16.1 ± 0.3	13 ± 1

### Identification of individual constituents of the EtOAc fraction from roots

The EtOAc fraction of roots showed the highest antibacterial and radical scavenging activities and the highest content of polyphenolic compounds, thus it was subjected to detailed chemical study. Seven individual compounds were isolated and their structures were elucidated by comparison of their spectral characteristics (^1^H and ^13^C NMR, MS) with literature data: tormentic acid **1** [25], 3-*O*-methylellagic acid-3′-*O*-*α*-3″-*O*-acetylrhamnopyranoside **2** [26], 3-*O*-methylellagic acid-3′-*O*-*α*-2″-*O*-acetylrhamnopyranoside **3** [26], catechin **4** [27], 3,3′-di-*O*-methylellagic acid-4-*O*-*β*-d-glucopyranoside **5** [28], niga-ichigoside F1 **6** [27], and gein **7** [27] (Spectral data of the isolated compounds are presented in Additional file 1).

### MIC of individual compounds from EtOAc fraction

Six isolated compounds from the EtOAc fraction of roots were subjected to MIC determination against *E. coli* SAIM WF+, *C. albicans* SAIM 562, *S. aureus* NBIMCC 3359 and *P. aeruginosa* NCTC 6749. Results are shown in Table 4. Tormentic acid had bacteriostatic activity against *C. albican*s SAIM 562 and *S. aureus* NBIMCC 3359 in concentrations, respectively, 500 µg/ml and 125 µg/ml. Catechin showed bactericidal effect against *S. aureus* NBIMCC 3359 and *P. aeruginosa* NCTC 6749 in concentrations, respectively, 250 and 500 µg/ml (Table 4). 3-*O*-methylellagic acid-3′-*O*-*α*-2″-*O*-acetylrhamnopyranoside **3** was isolated in very small amount (3.8 mg) and was not tested.

**Table 4:** Antibacterial activity of single compounds (mg/ml) isolated from *G. urbanum*

Compounds	Test bacteria			
	*S. aureus* 3359	*P. aeruginosa* 6749	*E. coli* WF+	*C. albicans* 562
Tormentic acid **1**	0.125*	> 2.5	> 2.5	0.5*
Catechin **4**	0.25**	0.5**	> 2.5	> 2.5
Gein **7**	> 2.5	> 2.5	> 2.5	> 2.5
Niga-ichigoside F1 **6**	> 2.5	> 2.5	> 2.5	> 2.5
3,3′-di-*O*-methylellagic acid-4-*O*-*β*-d-glucopyranoside **5**	> 2.5	> 2.5	> 2.5	> 2.5
3-*O*-methylellagic acid-3′-*O*-*α*-3″-*O*-acetylrhamnopyranoside **2**	> 2.5	> 2.5	> 2.5	> 2.5

2.5 mg/ml is the highest test concentration of compounds

* Bacteriostatic activity; ** bactericidal activity

## Discussion

The increasing antimicrobial resistance during the last decades drove scientist to search for new sources of antimicrobial compounds and more intensively to focus on investigation of various medicinal plants as an opportunity to deal with this burden. In our study, for the first time we explored the antimicrobial properties of the medicinal plant *G. urbanum.* Total MeOH extracts of roots and aerial parts of *G. urbanum* and their fractions obtained by subsequent extraction with petroleum ether, EtOAc and *n*-BuOH were studied.

The extracts and fractions obtained were investigated for antibacterial effect on a selected panel of Gram-positive and Gram-negative bacterial species. Our data showed that the response of the Gram-positive bacteria varied depending on the strains, wherein the growth of Gram-negative bacteria was not influenced by any of the tested extracts and fractions. The Gram-negative bacteria are considered to be more resistant due to their outer membrane and/or the presence of plasmid genes acting as a barrier to many environmental substances including antimicrobial agents [31]. Regarding the Gram-positive bacterial strains tested in our study, the four EtOAc and *n*-BuOH fractions showed stronger antibacterial activity than the total extracts or other fractions. The EtOAc fractions exerted the strongest antibacterial potential and the test strain *B. cereus* ATCC 9634 was found to be most sensitive. Lower concentration of EtOAc fractions from aerial parts (MIC 78 µg/ml) and roots (MIC 156 µg/ml) inhibited the visible growth of the test bacteria and suppressed their respiratory activity up to 26.2–28.9%, whereas higher concentrations (625 µg/ml) exerted bactericidal effect. The EtOAc and *n*-BuOH fractions were characterized by high polyphenolic content. Thus, it could be hypothesized that their antibacterial effect is most probably due to the adsorption of polyphenols to bacterial membranes with membrane disruption and subsequent leakage of cellular contents [32] and the generation of hydroperoxides from polyphenols [33].

Antioxidants are compounds involved in the defense mechanism of organisms against pathologies associated to the attack of free radicals [34]. These pathologies can lead to cancer, coronary heart disease, obesity, type 2 diabetes, hypertension, cataract, neurodegenerative diseases, including Alzheimer’s and Parkinson’s diseases [35]. In our study we used two complementary methods to detect the radical scavenging activity of extracts and fractions of *G. urbanum*: DPPH• assay based on electron transfer process, and superoxide anion radical scavenging assay based on a hydrogen atom transfer process [36]. The total MeOH extracts demonstrated some DPPH• scavenging activity. Among all the samples tested, EtOAc fractions from roots and aerial parts showed the highest DPPH• radical scavenging potential (with EC_50_ values of 0.8 and 1.5 µg/ml, respectively), even better than that of the positive control caffeic acid. In comparison, the study of Owczarek et al. [14] demonstrated that EtOAc fractions of wildly growing *G. urbanum,* collected from Lodz area, had EC_50_ values of 3.16 and 4.18 µg/ml for the root and aerial parts, respectively. The highest DPPH• radicals inhibition percentage was found for the EtOAc fractions which demonstrated also the highest total phenolic content (Table 3). This is an expected result, because the Folin–Ciocalteu method is based on an oxidation–reduction reaction and, as such, can be considered another method for antioxidant evaluation [37]. The *n*-BuOH fractions also demonstrated high antioxidant activity (about 80% of the EtOAc activity) (Fig. 2), although their phenolic content is lower (about 4 fold) as compared to the EtOAc. As is known, different phenolic compounds have different responses in the assay method [38]. Thus, *G. urbanum* MeOH extracts and fractions thereof (except petroleum ether fraction) contain phytochemical constituents that are capable of scavenging free radicals to prevent the potential damage.

As a complementary method for antiradical activity, the superoxide anion radical scavenging test was applied. Superoxide anion radical is one of the strongest agents damaging living cells, specifically because of its participation in the formation of more powerful and dangerous hydroxyl radicals as well as singlet oxygen, both of which contribute to oxidative stress [39]. The results of our study revealed that EtOAc and *n*-BuOH fractions of MeOH extracts had effective capacity of scavenging for superoxide radical (Fig. 3) Furthermore, superoxide scavenging activity was found to be high in EtOAc fractions in a concentration dependent manner. The superoxide scavenging activity correlated also with total phenolic content (Table 3), thus, suggesting its antioxidant potential. Recent studies have shown that polyphenols contribute significantly to the superoxide anion radical scavenging activity of medicinal plants [39, 40].

In general, our studies demonstrated that EtOAc fractions from aerial parts and roots from *G. urbanum* were characterized by the highest antibacterial and antiradical activity, and the highest amount of total phenolics. For this reason, we tried to isolate some individual constituents of the EtOAc fraction of the roots and evaluate their contribution to the observed activities. We isolated and identified 7 individual compounds from the EtOAc fraction of MeOH extract of *G. urbanum* roots. Two of them are the well known *G. urbanum* constituents, catechin **4** and gein **7**. In addition, we found two acetylated ellagic acid rhamnosides, new for the genus G*eum*—3-*O*-methylellagic acid-3′-*O*-*α*-3″-*O*-acetylrhamnopyranoside **2** and 3-*O*-methylellagic acid-3′-*O*-*α*-2″-*O*-acetylrhamnopyranoside **3**, and three compounds, new for the species *G. urbanum*: 3,3′-di-*O*-methylellagic acid-4-*O*-*β*-d-glucopyranoside **5** and the triterpenoids tormentic acid **1** and niga-ichigoside F1 **6**. The compounds isolated were tested for their antimicrobial activity (Table 4). Catechin **1** showed some activity against *S. aureus* and *P. aeruginosa*, while tormentic acid was active against *S. aureus* and *C. albicans.*

Catechin (flavan-3-ol) **4** [41] was isolated earlier from *G. urbanum* L. [12] and *G. iranicum* [27]. Catechin, isolated from the crude cinnamon stick extract was inactive against *B. cereus, L. monocytogenes, S. aureus, E. coli, Salmonella anatum* [42]. In contrast, a bactericidal effect of catechin against *S. aureus* and *P. aeruginosa* was observed in our study.

The pentacyclic triterpene tormentic acid **1** was isolated earlier from *G. rivale* [43] and *G. japonicum* [44]. This acid has demonstrated anticancer, anti-inflammatory and antiatherogenic properties [45–47] and potential in the prevention or treatment of atherosclerosis [48]. According to Jovel et al. [49] tormentic acid did not exhibit antibacterial activity against MRSA. In our study we found that this compound possessed bacteriostatic effect against *S. aureus* and antifungal activity against *C. albicans*.

The other isolated compounds were inactive against the test microorganisms in concentrations up to 2.5 mg/ml. Nevertheless, there are literature data claiming that some of them possess other useful activities.

Gein **7** is a phenolic glycoside from the group of phenylpropanoids [3]. It was isolated before from *G. iranicum* [27], *G. japonicum* [50] and *G. urbanum* [11]. So far, there is no evidence that it exhibits any antibacterial activity [27].

Niga-ichigoside F1 **6**, a triterpene glycoside [51], was isolated from *G. japonicum* [12, 50, 52] and *G. rivale* [43]. Cheng et al. [53] reported that this compound enhanced the efficacy of cardiogenic differentiation of endogenous bone marrow derived from mesenchymal stem cells. It possesses also anti-inflammatory and antinociceptive action [54].

3,3′-di-*O*-methylellagic acid-4-*O*-*β*-d-glucopyranoside **5** was isolated earlier from *G. japonicum* [1], but to the best of our knowledge, no literature data on biological activity of this compound are available.

The acetylated rhamnosides of *O*-methylellagic acid: 3-*O*-methylellagic acid-3′-*O*-*α*-3″-*O*-acetylrhamnopyranosidе **2** and 3-*O*-methylellagic acid-3′-*O*-*α*-2″-*O*-acetylrhamnopyranosidе **3** are known constituents of the stem bark of *Eucalyptus globulus* but have not been identified in the genus *Geum* so far. They were found to inhibit lipid peroxidation in rat liver microsomes [26]. In general, ellagic acid rhamnoside derivatives are known to inhibit *S. aureus* biofilm formation and improve response to antibiotics [55].

It is known from the literature that often the components in crude extracts or fractions demonstrate high antimicrobial activities when they are applied together as part of the mixture which points to synergistic interactions [56]. Our results suggest that this could be the case with *G. urbanum.*

## Conclusion

This study showed that *Geum urbanum* L. has antimicrobial potential against Gram-positive bacteria and high free radical scavenging activity. Ethyl acetate seems to be the best solvents to concentrate antimicrobial and antioxidant compounds from MeOH extracts of the investigated plant, which confirms previous studies [14]. In addition, individual compounds with biological potential were isolated from the EtOAc root fraction, some of which were found for the first time in the genus *Geum* and in the species *Geum urbanum* L. Our results reveal that *G. urbanum* L. is a perspective medicinal plant and deserves further, more detailed studies.

## Additional file

**Additional file 1: Figure S1.** ^1^H and ^13^C NMR spectra of tormentic acid (1) in Pyridine d_5_. **Figure S2.** ^1^H, ^13^C, HSQC and HMBC NMR spectra of 3-*O*-methylellagic acid-3′-*O*-*α*-3″-*O*-acetylrhamnopyranoside (2) in CDCl_3_:CD_3_OD 1:1. **Figure S3.** ^1^H NMR spectrum of 3-*O*-methylellagic acid-3′-*O*-*α*-2″-*O*-acetylrhamnopyranoside (3) in CDCl_3_:CD_3_OD 1:1. **Figure S4.** ^1^H NMR spectrum of cathechin (4) in CD_3_OD. **Figure S5.** ^1^H, HSQC and HMBC NMR spectra of 3,3′-di-*O*-methylellagic acid-4-*O*-*β*-d-glucopyranoside (5) in DMSO. **Figure S6.** ^1^H and ^13^C NMR spectra of niga-ichigoside F1 (6) in CD_3_OD. **Figure S7.** ^1^H spectrum of gein (7) in CD_3_OD.

## Abbreviations

MeOH: methanol
EtOAc: ethyl acetate
*n*-BuOH: *n*-butanol
NBIMCC: National Bank for Industrial Microorganisms and Cell Cultures, Bulgaria
ATCC: American Type Cell Culture Collection, USA
SAIM: Collection of the Stephan Angeloff Institute of Microbiology, Bulgaria
NCTC: National Collection of Type Cultures, England
MHA: Muller Hinton agar
MHB: Muller Hinton broth
MIC: minimal inhibitory concentration
MBC: minimal bactericidal concentration
MTT: 3-(4,5-dimethylthiazolyl-2)-2,5-diphenyltetrazolium bromide
DEHA: dehydrogenase activity
DPPH: 1,1-diphenyl-2-picrylhydrazyl
NBT: nitro blue tetrazolium
CC: column chromatography
TLC: thin-layer chromatography
NMR: nuclear magnetic resonance spectroscopy
CFU: colony-forming units
