# Supplementary material for: Antimicrobial and antioxidant potential of different solvent extracts of the medicinal plant Geum urbanum L
Source: Chem Cent J. 2017 Nov 7;11:113. doi: 10.1186/s13065-017-0343-8 (PMC5676587; doi:10.1186/s13065-017-0343-8)

**Fig S1. ^1^H and** **^13^C NMR spectra of tormentic acid (1) in Pyridine d_5_.**


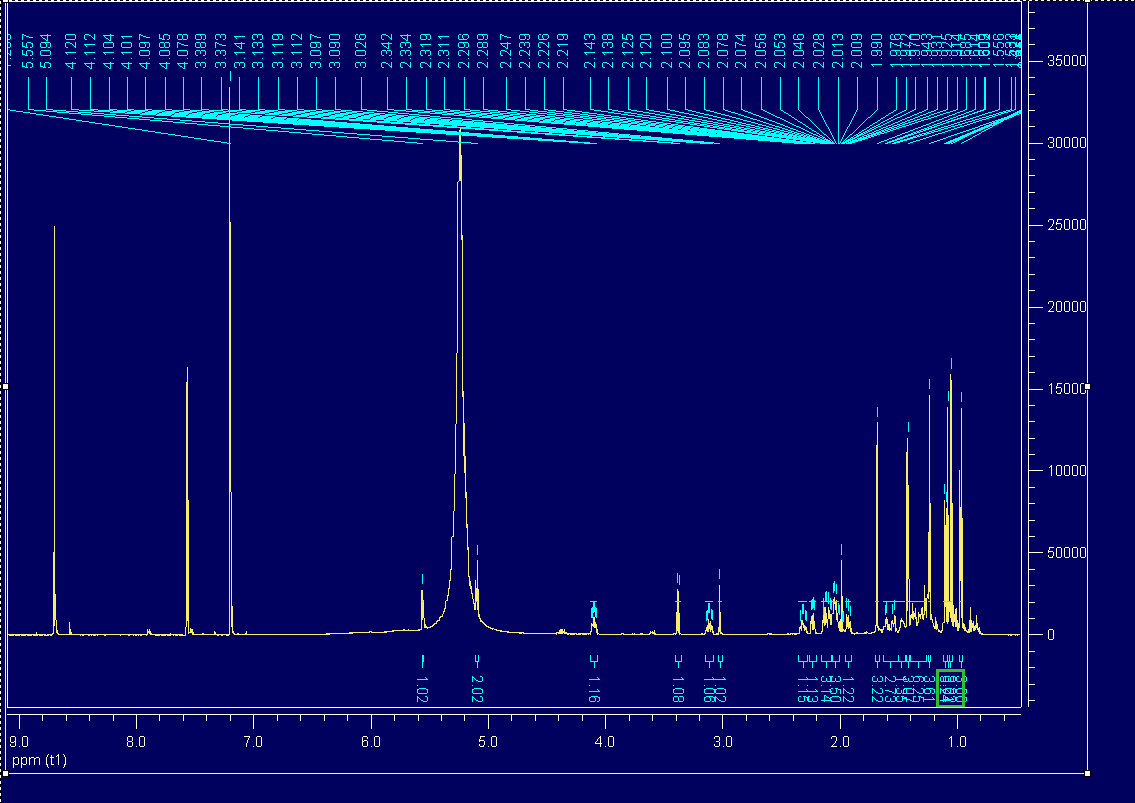


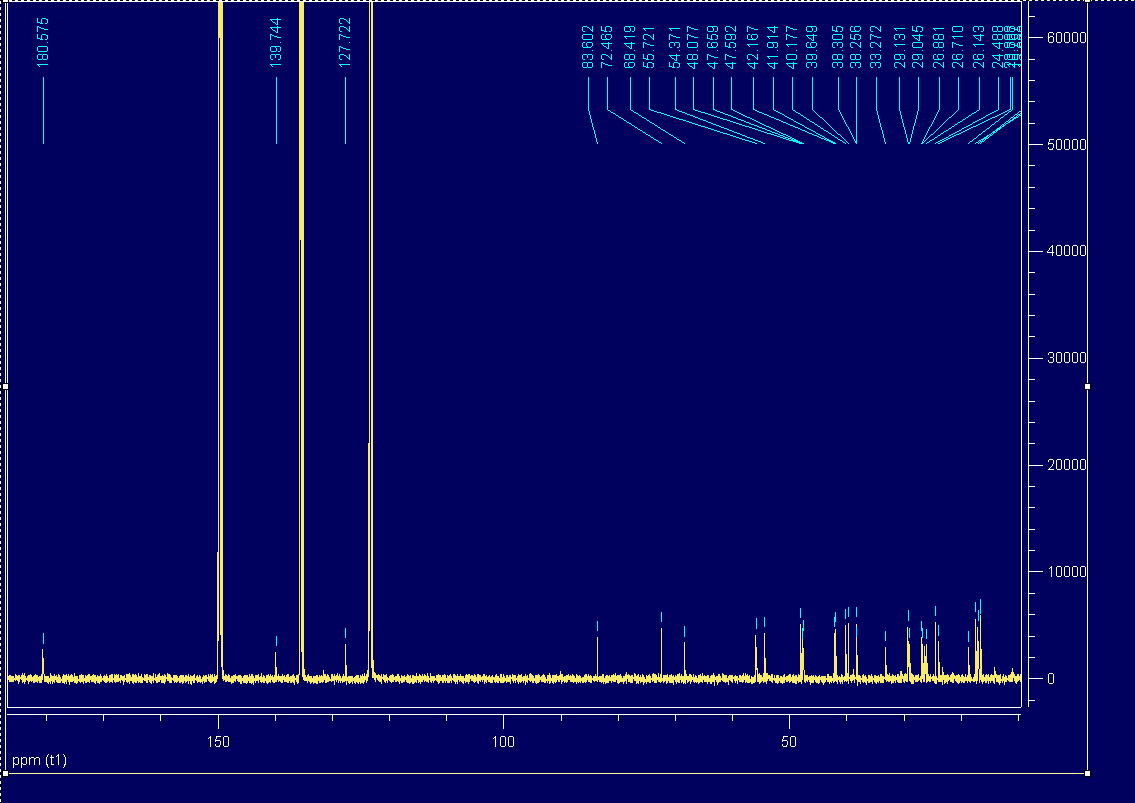


**Fig S2. ^1^H, ^13^C, HSQC and HMBC NMR spectra of 3-*O*-methylellagic acid-3’-*O*-*α*-3’’-*O*-acetylrhamnopyranoside (2) in CDCl_3_:CD_3_OD 1:1.**


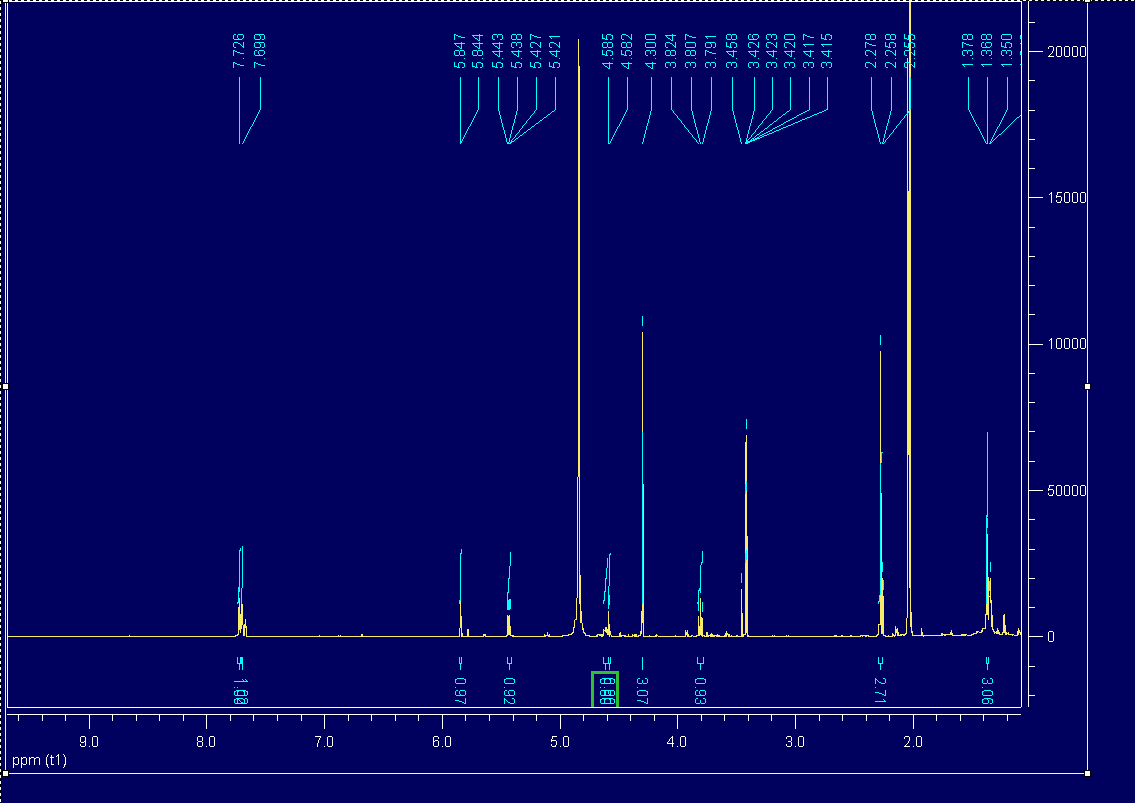


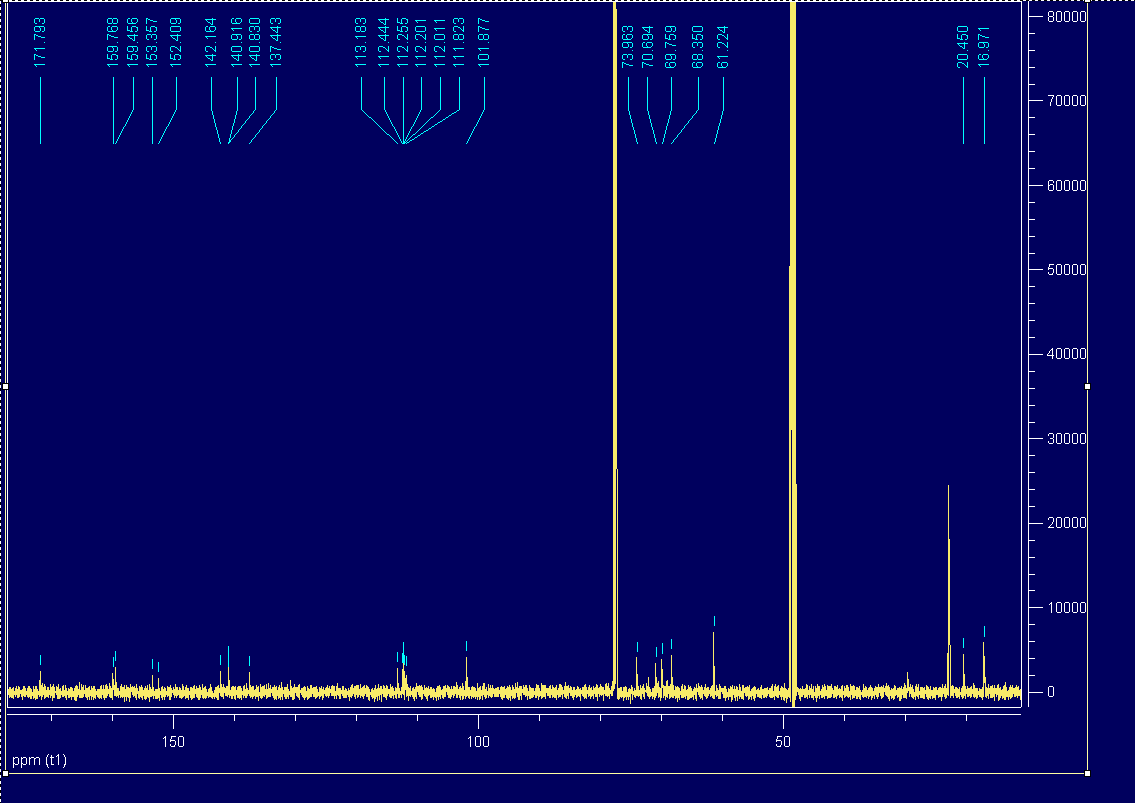


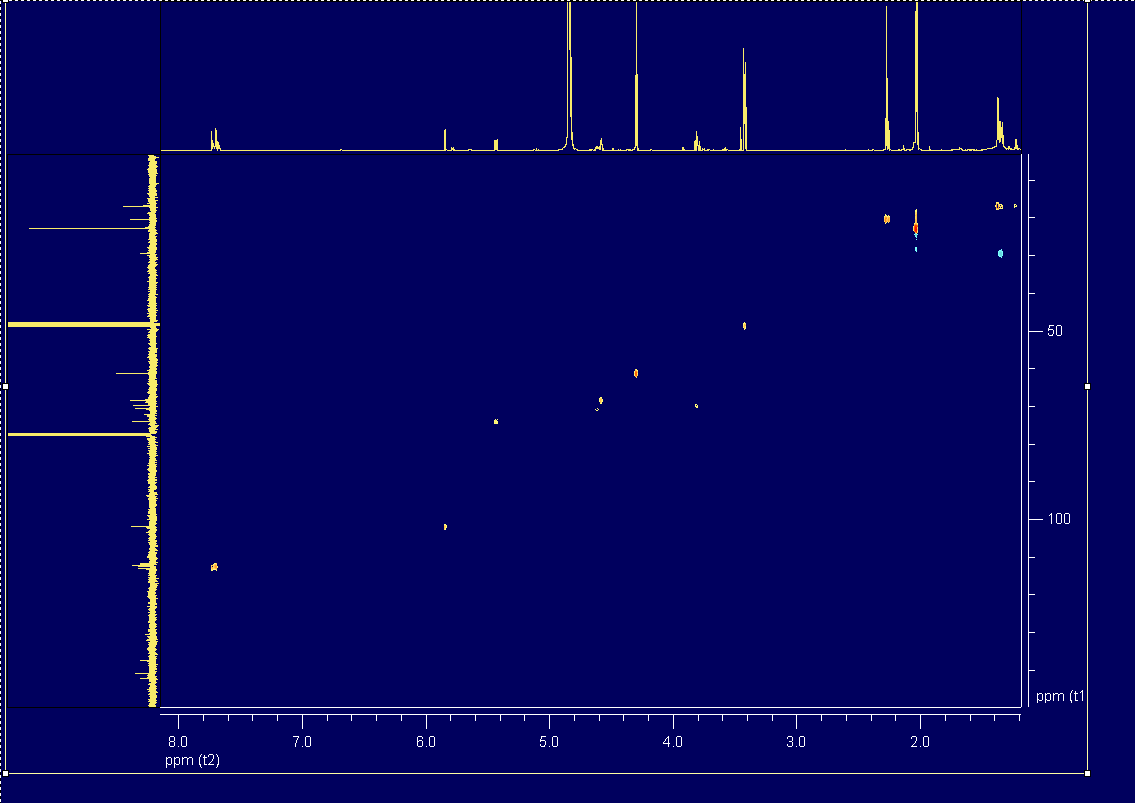


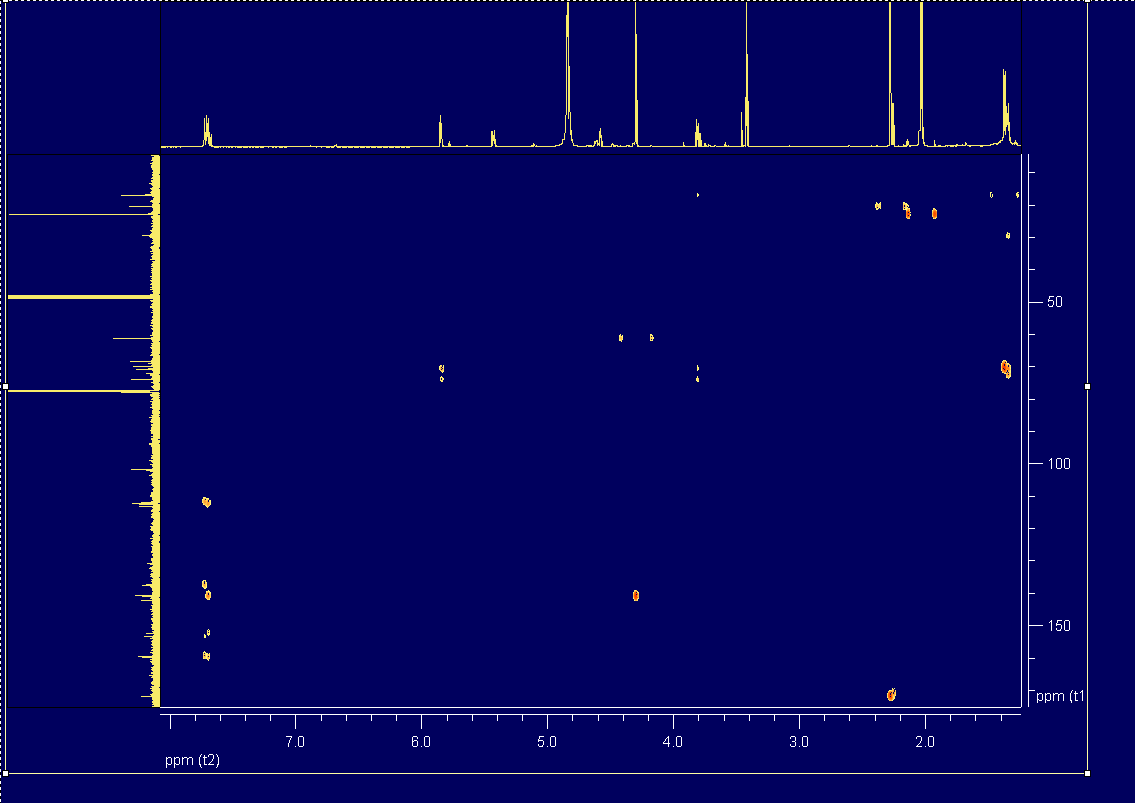


**Fig S3. ^1^H NMR spectrum of 3-*O*-methylellagic acid-3’-*O*-*α*-2’’-*O*-acetylrhamnopyranoside (3) in CDCl_3_:CD_3_OD 1:1.**


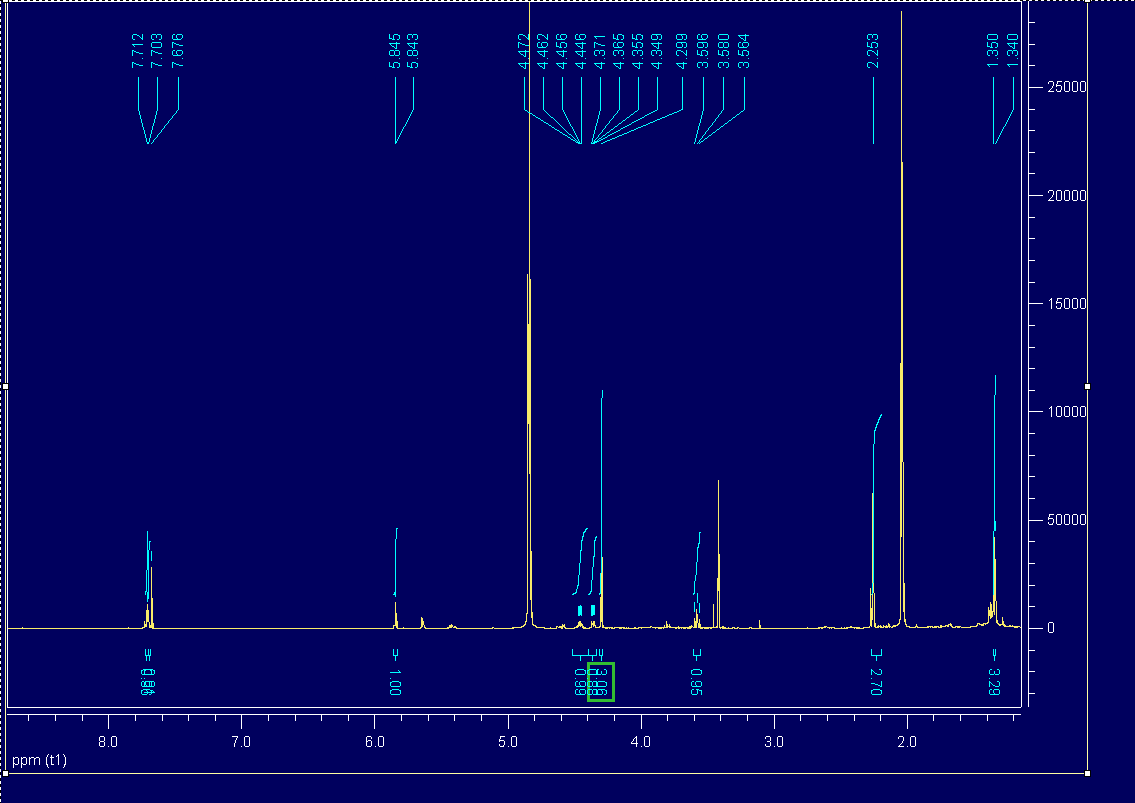


**S4. Fig. ^1^H NMR spectrum of cathechin (4) in CD_3_OD.**


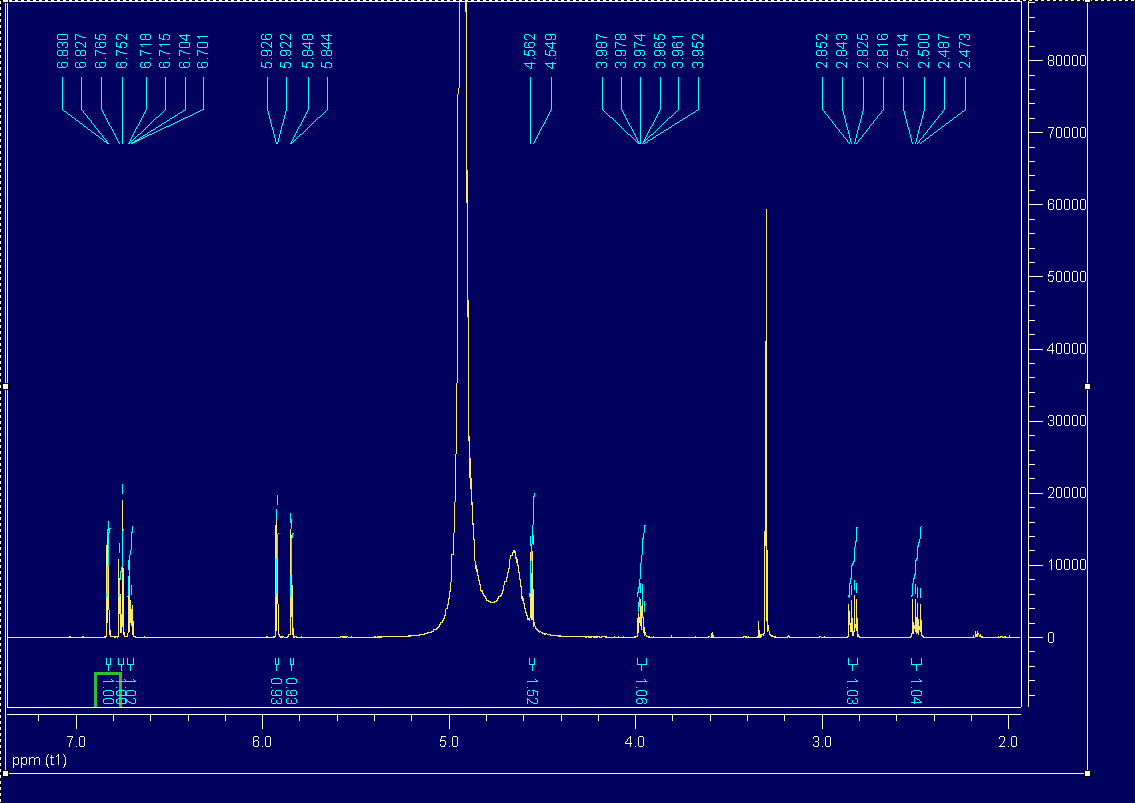


**Fig S5. ^1^H, HSQC and HMBC NMR spectra of 3,3’-di-*O*-methylellagic acid-4-*O*-*β*-d-glucopyranoside (5) in DMSO.**


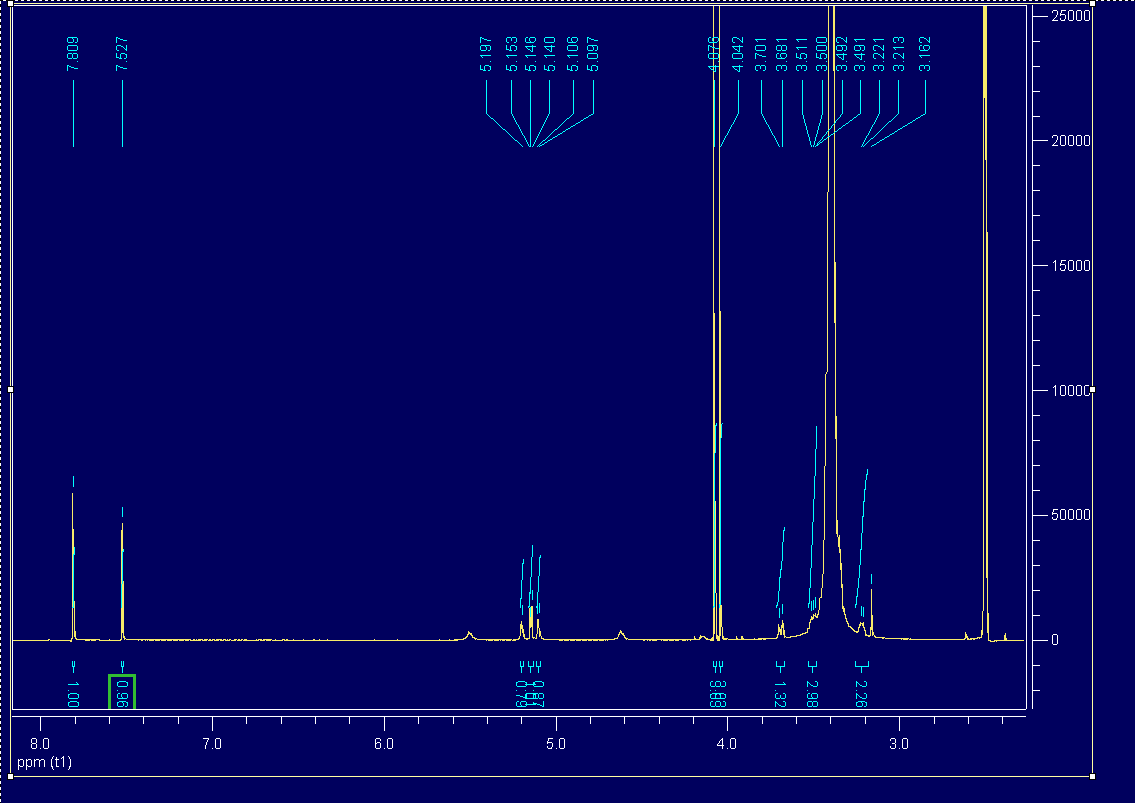


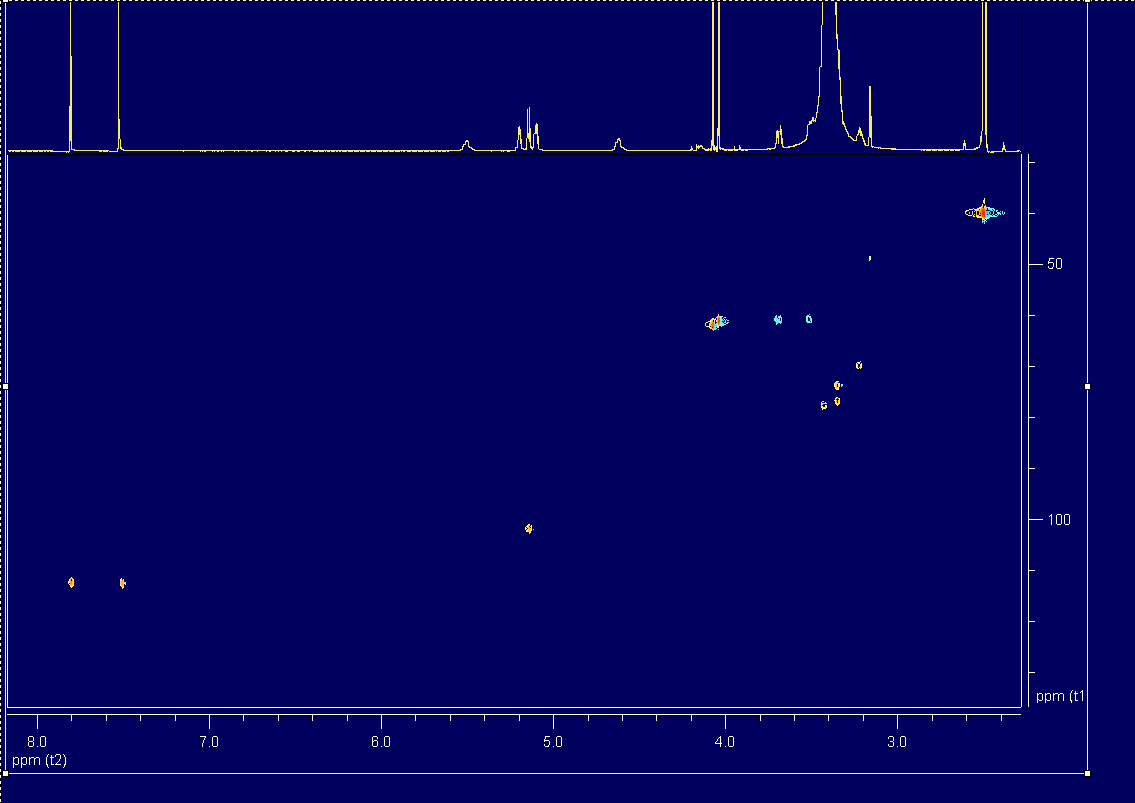


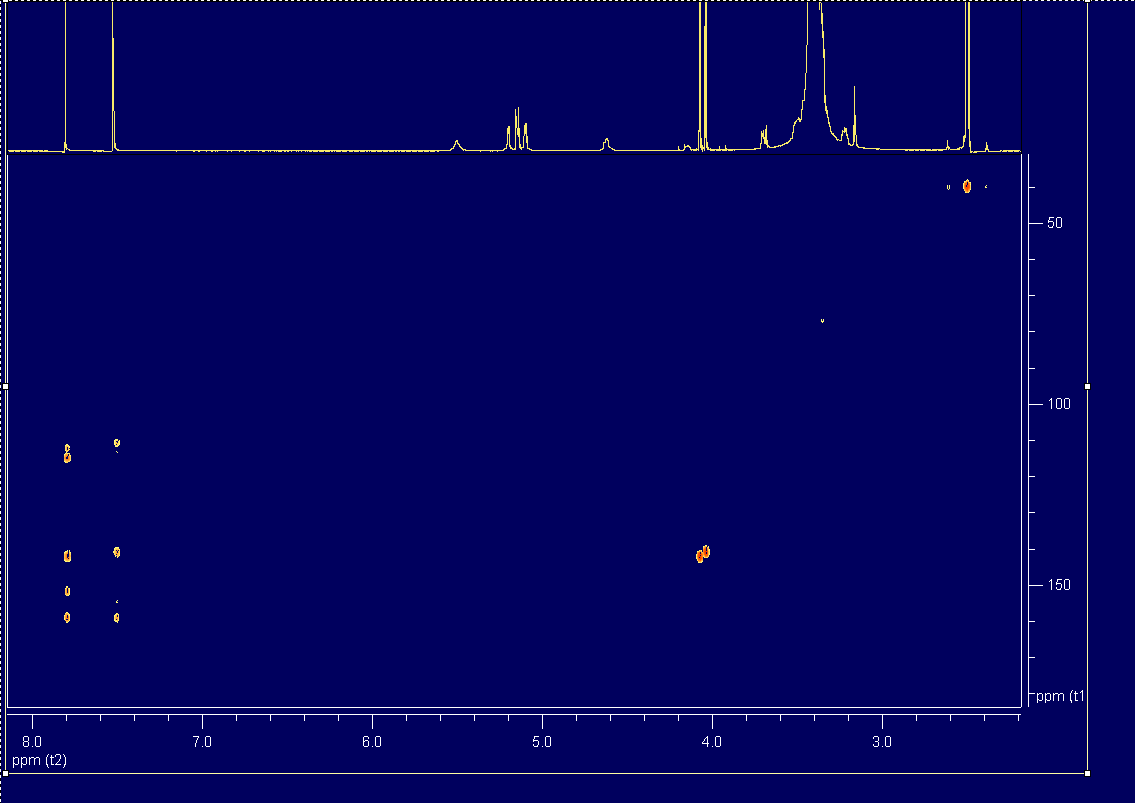


**Fig S6. ^1^H and ^13^C NMR spectra of niga-ichigoside F1 (6) in CD_3_OD.**


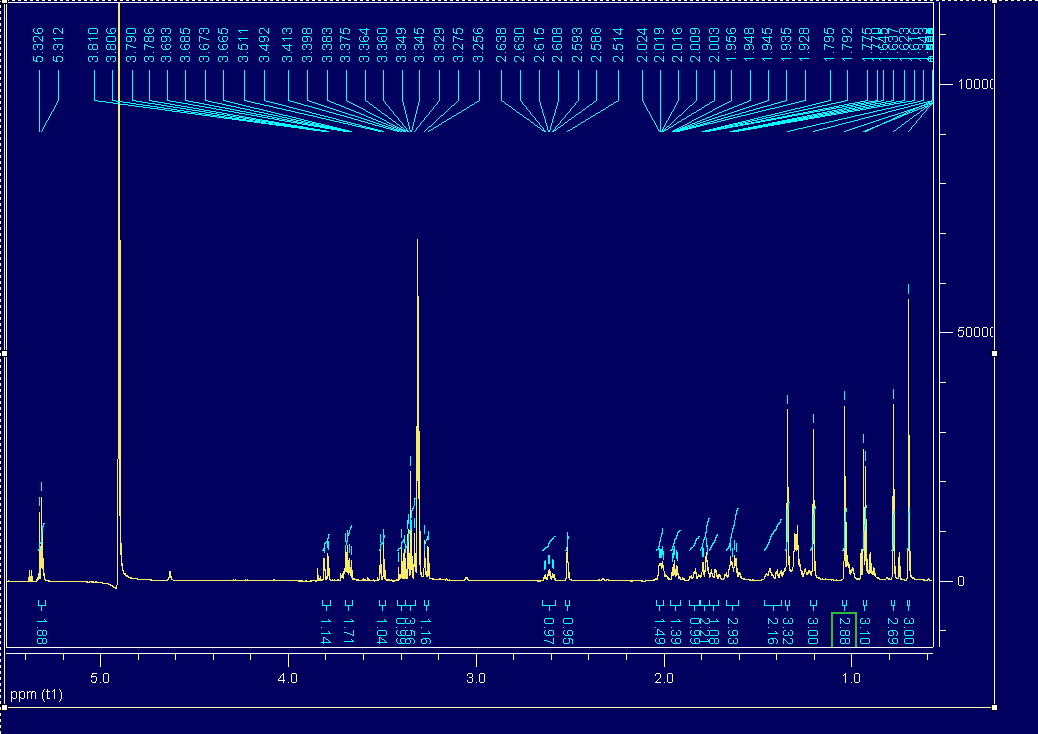


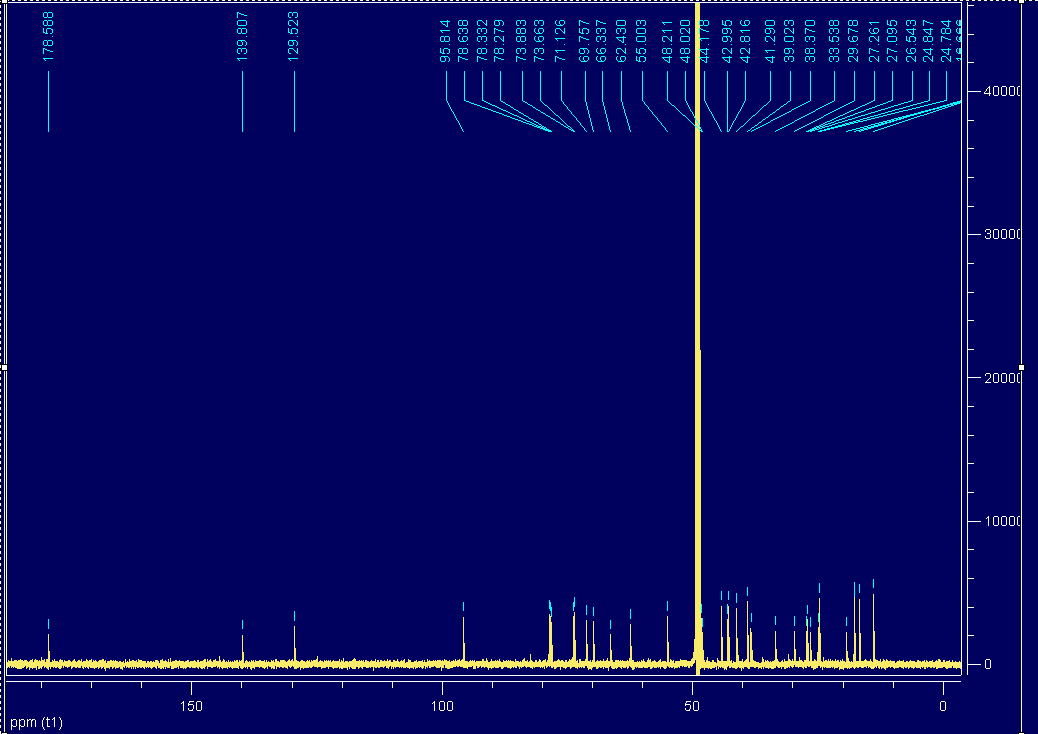


**Fig S7. ^1^H spectrum of gein (7) in CD_3_OD.**


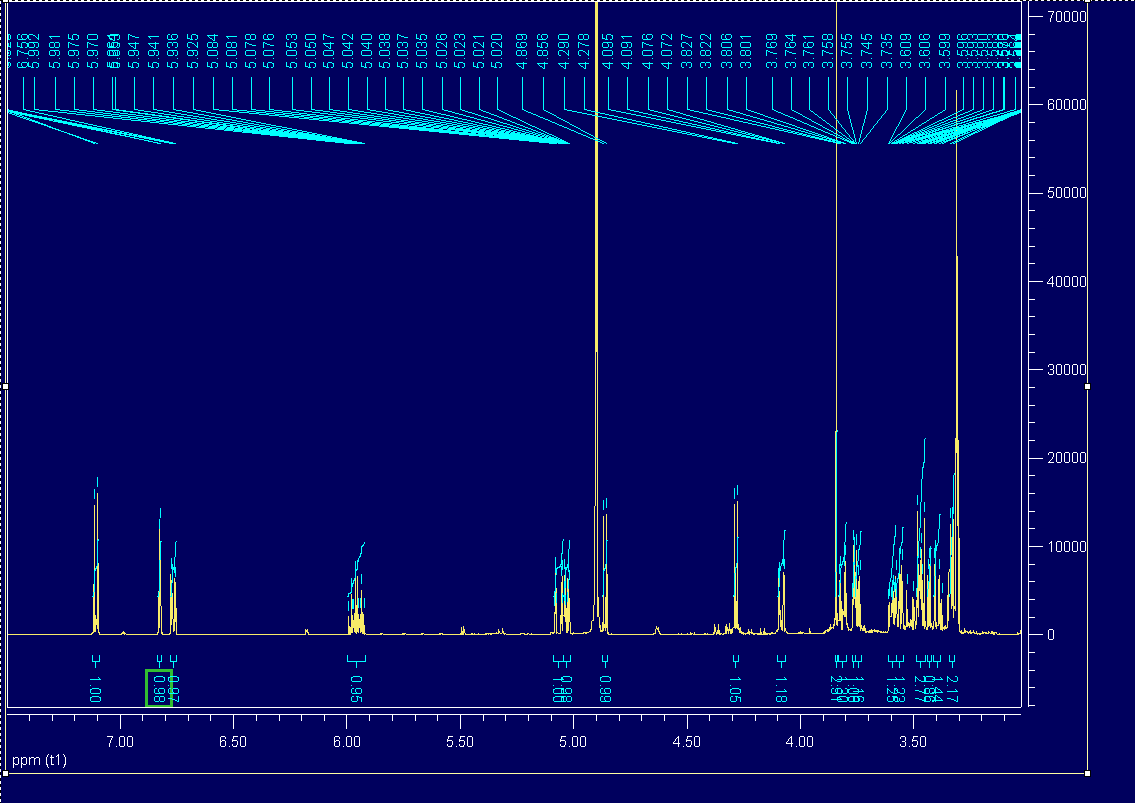

Supplement: Supplementary file 1 — Additional file 1: Figure S1. 1H and 13C NMR spectra of tormentic acid (1) in Pyridine d5. Figure S2. 1H, 13C, HSQC and HMBC NMR spectra of 3-O-methylellagic acid-3′-O-α-3″-O-acetylrhamnopyranoside (2) in CDCl3:CD3OD 1:1. Figure S3. 1H NMR spectrum of 3-O-methylellagic acid-3′-O-α-2″-O-acetylrhamnopyranoside (3) in CDCl3:CD3OD 1:1. Figure S4. 1H NMR spectrum of cathechin (4) in CD3OD. Figure S5. 1H, HSQC and HMBC NMR spectra of 3,3′-di-O-methylellagic acid-4-O-β-d-glucopyranoside (5) in DMSO. Figure S6. 1H and 13C NMR spectra of niga-ichigoside F1 (6) in CD3OD. Figure S7. 1H spectrum of gein (7) in CD3OD. [file 13065_2017_343_MOESM1_ESM.doc]
